# Local Investigators Significantly Overestimate Overall Response Rates Compared to Blinded Independent Central Reviews in Uncontrolled Oncology Trials: A Comprehensive Review of the Literature

**DOI:** 10.3389/fphar.2022.858354

**Published:** 2022-05-16

**Authors:** Cinzia Dello Russo, Pierluigi Navarra

**Affiliations:** ^1^ Section of Pharmacology, Department of Healthcare Surveillance and Bioethics, Fondazione Policlinico Universitario A. Gemelli IRCCS, Università Cattolica Del Sacro Cuore, Rome, Italy; ^2^ MRC Centre for Drug Safety Science and Wolfson Centre for Personalized Medicine, Institute of Systems Molecular and Integrative Biology (ISMIB), University of Liverpool, Liverpool, United Kingdom

**Keywords:** objective response rate, ORR, progression-free survival, PFS, local investigators, BICR, oncology, pharmacotherapy

## Abstract

Several drugs gained market authorization based on the demonstration of improved progression-free survival (PFS), adopted as a primary endpoint in Phase 3 clinical trials. In addition, an increasing number of drugs have been granted accelerated approval, and sometimes regular approval, by the main regulatory agencies based on the evaluation of the overall response rate in Phase 1 and 2 clinical trials. However, while the overall survival is an unbiased measure of drug efficacy, these outcomes rely on the assessment of radiological images and patients’ categorization using standardized response criteria. The evaluation of these outcomes may be influenced by subjective factors, particularly when the analysis is performed locally. In fact, blinding of treatment is not always possible in modern oncology trials. Therefore, a blinded independent central review is often adopted to overcome the problem of expectation bias associated with local investigator assessments. In this regard, we have recently observed that local investigators tend to overestimate the overall response rate in comparison to central reviewers in Phase 2 clinical trials, whereas we did not find any significant evaluation bias between local investigators and central reviews when considering progression-free survival in both Phase 2 and 3 trials. In the present article, we have tried to understand the reasons behind this discrepancy by reviewing the available evidence in the literature. In addition, a further analysis of Phase 2 and 3 clinical trials that included the evaluation of both endpoints showed that local investigators significantly overestimate overall response rates compared to blinded independent central reviews in uncontrolled oncology trials.

## Introduction

Cancer is one of the major causes of death across the world, leading to a significant reduction of life expectancy in several countries. The burden of cancer is steadily increasing worldwide (Sung et al., 2021); in 2020, there were 19.29 million new cancer cases, 9.96 million cancer deaths, and a total of 50.55 million people living with cancer within 5 years of diagnosis (Global Cancer Observatory, 2020). The COVID-19 pandemic has negatively impacted on several clinical procedures in oncology, such as reduced access to screening programs, delayed diagnosis, and disruption in treatment schedules, possibly contributing to such increase. The rising incidence of cancer coupled with the high mortality rates highlights the need for new and more effective treatments in oncology. However, the outcome of anticancer treatments significantly depends on the extent of tumor disease at treatment initiation, which further highlights the relevance of preventive strategies as well as diagnostic procedures or other clinical interventions to allow early diagnosis of cancer and timely treatment initiation (Kwong et al., 2021; Okoli et al., 2021). With respect to the development of novel anticancer drugs, there is a continuous effort to define the most accurate methods as well as the relevant endpoints to assess the clinical benefit of new compounds in the context of different tumors (Daniele et al., 2020).

In this regard, the overall survival (OS) is an unbiased measure of drug efficacy, and thus it is considered the gold standard endpoint for most types of tumors (Delgado and Guddati, 2021). However, it is not always possible—or convenient—to take the OS as the primary endpoint of drug efficacy in clinical trials (Lebwohl et al., 2009). In fact, several drugs gained market authorization based on the demonstration of improved progression-free survival (PFS), which was adopted as a primary endpoint in Phase 3 clinical trials (Robinson et al., 2014). This is particularly frequent in the context of metastatic disease, although the PFS is not always predictive of beneficial effects in terms of OS (Haslam et al., 2019; Pasalic et al., 2020). Moreover, an increasing number of drugs have been granted accelerated approval, and sometimes regular approval, by the FDA based on the evaluation of the overall response rate (ORR) in Phase 1–2 clinical trials (Chen et al., 2019), although a large meta-analysis showed poor correlation between ORR and OS or PFS (Cooper et al., 2020). In addition, it has become progressively clear, especially with the development of targeted therapies, that not all new drugs cause shrinking of tumors, with disease stabilization observed in several cases. Therefore, it is also often necessary to perform evaluations of time-to-event endpoints during the early phases of clinical development (Gravanis et al., 2014).

Notably, both the ORR and the PFS rely on the assessment of radiological images and patients’ categorization based on standardized response criteria (Delgado and Guddati, 2021). The most frequently adopted criteria are the response evaluation criteria in solid tumors (RECIST) (Therasse et al., 2000; Eisenhauer et al., 2009), which have been adequately modified over time to better assess responses in specific clinical settings, for a specific tumor, or in relation to specific drugs, that is, immune check point inhibitors (Aykan and Özatlı, 2020). A major drawback is that the evaluation of these endpoints may be influenced by subjective factors, such as differences in tumor measurement, differences in the selection of target lesions, failure to diagnose new lesions, and differences in the analysis of non-target lesions (Amit et al., 2010; Iannessi et al., 2021). This may be particularly relevant when the evaluations are performed locally, considering that blinding of treatment is not always possible in modern oncology trials. Therefore, a blinded independent central review (BICR) is often adopted to overcome the problem of expectation bias associated with local investigator (LI) assessments (Dodd et al., 2008; Amit et al., 2010). When BICR is implemented, all the radiological images and selected clinical data acquired in the clinical trials are reviewed by independent radiologists who are blinded to treatment assignment and to any kind of clinical data that can influence the independent review process (Ford et al., 2009). In this regard, we have recently observed that LIs tend to overestimate the ORR in comparison to BICR in Phase 2 clinical trials, whereas we did not find any significant evaluation bias between LIs and BICR when considering PFS in both Phase 2 and 3 trials (Dello Russo et al., 2021; 2020). In the present article, we have tried to understand the reasons behind this discrepancy by reviewing the available evidence in the literature. In addition, we performed a further analysis of Phase 2 and 3 clinical trials that included the evaluation of both endpoints, ORR and PFS.

## Local Investigator Evaluation *Versus* Blinded Independent Central Review in the Assessment of Relevant Primary Outcomes

### Overall Response Rate

The ORR measures the response to a pharmacological treatment through the evaluation of changes in the overall tumor burden in comparison to baseline values (Aykan and Özatlı, 2020). In solid tumors, this is calculated using RECIST criteria (Therasse et al., 2000), updated in 2009 as RECIST v1.1 (Eisenhauer et al., 2009). These criteria allow to classify patients into different groups considering the effects of the investigational drug on the tumor lesions. In brief, patients are categorized as having a complete response (CR), partial response (PR), stable disease (SD), or progressive disease (PD). The ORR is then calculated as the percentage of patients with CR and PR over the total number of eligible patients, that is, all subjects included in the trial (Eisenhauer et al., 2009). Response evaluation is usually based on arbitrary cutoff values at a specific time point, usually at 4 or 8 weeks after treatment initiation (Eisenhauer et al., 2009; Aykan and Özatlı, 2020).

In the first analysis of 18 Phase 3 clinical trials, a significant variability was reported between the LI assessments, and the BICR results in the evaluation of the tumor response rate (RR) (Tang et al., 2010). Despite using a general abbreviation, in the majority of the analyzed trials, the RR is indeed equal to the ORR, as per the abovementioned definition. However, in two trials, CR was observed only in one enrolled patient, which leaves the comparison among LI and BICR to the evaluation of the rate of PRs (Escudier et al., 2007; Motzer et al., 2007). In 14 out of 18 trials, the RR values measured by LIs were consistently greater than those by BICR for both the experimental and the control arms of the studies. For the remaining four trials, in one study, the evaluation of the RR was higher in the experimental group by LIs than that by BICR and lower for the control arm; in two trials, the RR was reported to be inferior by LIs in comparison to BICR in the experimental group and superior in the Control arm; and in one trial, the RR was consistently lower in both arms of the study by the LI in comparison to BICR. The analysis was performed by comparing the absolute values reported for the RR. Therefore, the RR reported by LIs was greater in 31 out of 36 evaluations, with an estimated mean difference of +4.57% (95% confidence interval, CI, 2.95–6.19%), thus suggesting that overall the LIs were more “optimistic” than the independent reviewers in the evaluation of tumor RR. These results are consistent with a more recent analysis carried out by our research group on data from 20 Phase 2 clinical trials having the ORR as primary or secondary endpoint, assessed by both LIs and through BICR (Dello Russo et al., 2021). In some trials, more than one treatment group was included for a total of 33 ORR values. In most cases (26/33, 78.8%), the LI assessment was more “optimistic” in the evaluation of ORR, with an average of + 17.5% positive responses than the central independent reviewer. Most trials had an open-label design, and 12 out of 20 trials (60%) had a single group assignment. When parallel groups were included, these referred, for example, to different doses of the same drugs (NCT02094573, brigatinib 90 mg, and brigatinib 90–180 mg) (Kim et al., 2017) or to the evaluation of the same drug in different cohorts of patients (NCT02336451, ceritinib in different cohorts of patients with brain metastases, no evidence of leptomeningeal carcinomatosis, and exposed to different previous therapies) (Chow et al., 2019). Therefore, most trials lacked a comparator arm. Our data are however consistent with previous evidence from the evaluation of 28 Phase 3 clinical trials, six of which were carried out according to a blinded design (Zhang et al., 2017). In this study, with respect to the analysis of ORR on both the experimental and control arms of the studies, the authors found a lower response frequency in the central review compared with LI assessment. The discrepancy was larger in the open-label trials, for a positive primary outcome, and central assessed primary endpoint. However, this phenomenon impacted both the experimental and the comparator arm; therefore, it did not cause any significant evaluation bias between the two reviews. The ratio of odd ratios (ORs) was 1.02, with a 95% CI of 0.97–1.07 (*p* = 0.42), thereby including the value of 1 and suggesting a high degree of concordance between the two assessments. Interestingly, 16 trials reported data on the disease control rate (DCR) with similar results to those observed for the ORR (Zhang et al., 2017). Consistently, in a subsequent large analysis of 76 Phase 3 clinical trials, including over 45,000 patients, the authors found eight trials with discrepancy in the evaluation of ORR among LIs and BICR. In seven of these trials, ORR was included as a secondary endpoint, further indicating that the ORR is rarely used as primary endpoint in Phase 3 clinical trials. Interestingly, in five out of eight trials, the BICR appeared to be more positive than the LI assessments (please refer to Table 2 in the original paper, Zhang et al. (2018)). The ORR was included as a primary endpoint in a clinical trial investigating the potential beneficial effect of peptide vaccine therapy as add-on treatment to aldesleukin (IL-2) in patients with locally advanced or metastatic melanoma (NCT00019682, Schwartzentruber et al., 2011). Interestingly, in this study, the BICR was more optimistic in the evaluation of the ORR in comparison to LI, although both evaluations found the addition of the vaccine more effective than the treatment with aldesleukin alone. Interestingly, the vaccine significantly increased the PFS and OS (although the study was not powered to assess these outcomes), indicating in this case a correlation between ORR and the other outcomes. However, the pooled analysis carried out on 29 trials that included the evaluation of ORR showed no significant differences among the two reviews (Zhang et al., 2018). Taken together, these data, therefore, suggest that there is an overall trend to overestimate the ORR by LIs. However, in controlled studies no significant differences can be found in the analysis of the beneficial effects of the investigational drug versus the comparator treatments among the two reviews, thus highlighting the reliability of the LIs in the evaluation of this endpoint in Phase 3 clinical trials.

### Progression-Free Survival

The PFS is defined as the time from randomization or treatment initiation until first evidence of objective tumor progression or death from any cause (Lebwohl et al., 2009; Amit et al., 2011; Delgado and Guddati, 2021). It is a time-to-event endpoint based on the assessment of disease progression at different time points defined in each clinical trial. It depends on tumor growth. This evaluation usually requires shorter time and a smaller population than OS, and it is not affected by subsequent therapies. Thus, it is often used as a primary endpoint in Phase 3 clinical trials, especially in the context of advanced/metastatic disease (Lebwohl et al., 2009). However, the data obtained may be subject to measurement error and bias since progression is defined based on standard criteria and is not objective as the time of death (Amit et al., 2010). That is why BICR is often recommended to minimize possible investigator bias, except when the trial is truly double-blinded (Amit et al., 2010). However, also in these cases, the occurrence of adverse drug effects can be valuable information for the investigator to reveal the treatment arm in which the patient is possibly allocated (Dodd et al., 2008).

In an initial analysis of concordance among LIs and BICR, data from 7 Phase 3 trials showed that there were not significant differences in the assessment of PFS and treatment efficacy among the two reviews (Dodd et al., 2008). However, in a subsequent study, a certain degree of variability between the LI assessments and the BICR results with respect to the evaluation of PFS was found in eight Phase 3 clinical trials (Tang et al., 2010). This analysis focused mainly on differences in the median times, showing that the estimated mean difference in PFS was ≥0.19 (95% CI, −0.68 to +0.29) months. The concordance of treatment effect, as measured by the hazard ratio (HR) for LIs and BICR, could be analyzed only in four trials, two of which reported differences among the LIs and BICR (Tang et al., 2010). In contrast, a large meta-analysis, promoted by several pharmaceutical companies, namely, GlaxoSmithKline, AstraZeneca, Eli Lilly, and Hoffman LaRoche, and carried out on 27 Phase 3 trials with PFS, showed strong correlation (*r* = 0.947) between the LI assessments and BICR. The estimated ratio of HRs between LIs and BICR was 1.03 (95% CI, 0.98–1.08), implying approximately 3% difference between the two evaluations (Amit et al., 2011). However, in a subsequent analysis of 76 Phase 3 clinical trials, 10 trials were identified with inconsistencies between the two reviews with respect to the assessment of PFS (Zhang et al., 2018). These studies mostly included PFS as the primary endpoint. In seven out of 10 trials, the LIs reported lower HR values in comparison to BICR, which means a larger beneficial effect of the investigational drug estimated locally. Consistent with data on the ORR discussed above, the pooled analysis carried out on 72 trials that included the evaluation of PFS showed no significant differences among the two reviews (please refer to Table 3 in the original paper, Zhang et al. (2018)). In a recent analysis performed by our group on 28 Phase 3 clinical trials, we compared the HRs for PFS between LIs and BICR calculating a discrepancy index through the ratio of the HRs among the two reviews, and we did not find any significant evaluation bias among the two assessments (Dello Russo et al., 2020). The estimated mean discrepancy index was 0.98 (95% CI, 0.927–1.032). We also performed subsequent analyses, dividing the trials by year, by tumor type, by drug type, and by study design. Interestingly, the discrepancy index did not include the value 1 only in the subgroup of trials started in the period 2003–2006, with the blinded assessment showing increased benefit than the LI assessment. We can hypothesize that the improvement in the diagnostic skills in clinical trials and the continuous refinement of diagnostic criteria per different tumors has progressively contributed to the reduction of evaluation bias. Consistently, we found a relatively higher variability (large CI) in trials testing biological agents, which was mainly due to the high discrepancy recorded in trial KEYNOTE-61 testing the effects of the immune check point inhibitor pembrolizumab (Shitara et al., 2018). In this regard, it is now well established that standard RECIST criteria cannot be applied to the assessment of immunotherapy efficacy in solid tumors and needed to be modified for these drugs, so-called immune-related response evaluation criteria in solid tumors (iRECIST) (Seymour et al., 2017). No other relevant differences were found in the subgroup analysis based on tumor type (trials in onco-hematology and solid tumors) and drug type (trials testing small-molecules and those testing biologicals). We also found a trend toward more optimistic assessments by the independent review in double-blinded trials. Taken together, these data suggest that the two approaches, local and central review, with respect to PFS, lead to substantially similar results. These results further support the notion that the BICR is not necessary for all clinical trials, but it can be considered based on specific cases or carried out as an audit on a subset of enrolled patients (Amit et al., 2010).

## Additional Evidence on the Comparison Between Local Investigators and Independent Reviewers

In our previous analysis, we found that the LIs tend to overestimate the ORR in comparison to BICR, whereas high concordance was observed for the evaluation of PFS. This result was unexpected, considering that both endpoints rely on the same criteria to assess the radiological images as well as to establish response to therapy and/or disease progression. In an attempt to clarify the reasons behind the differences between LIs and BICR in relation to the evaluation of these two outcomes, we accessed the clinicaltrials.gov database and the clinicaltrialsregister.eu (EudraCT) database on 30 October 2021 and searched for clinical trials that included both the ORR and the PFS as endpoints and evaluations by both LIs and blinded assessors. We have already analyzed a subgroup of Phase 2 clinical trials that included both the ORR and the PFS, showing no differences between local and central reviewers with respect to PFS (Dello Russo et al., 2021). We aimed to update and extend this initial analysis by including also the Phase 3 clinical trials.

### Research Strategy

The research strategy was based on the methodological approach previously described (Dello Russo et al., 2020). In brief, in the clinicaltrials.gov database, we searched for clinical trials selected by the search string ((investigator-assessed) OR and (investigator-assessment) AND (Cancer) and posted in the database within the data-lock date of 30 October 2021. We found a total of 1389 interventional clinical trials, of which 301 Phase 2 studies with results and 171 Phase 3 studies with results. Among these, we then selected for the analysis clinical trials that included both the ORR and the PFS evaluated both locally and by BICR. According to these inclusion criteria, we selected 17 Phase 2 and 18 Phase 3 clinical trials. However, two Phase 2 clinical trials were subsequently excluded, namely, the NCT01258608 trial, because the result section only included ORR evaluated by BICR (64.4 months, 95% CI, 48.80–78.10) and the NCT02108964 clinical trial for which no results were posted in the database at the data-lock date.

A further search was carried out in the EudraCT database using the same search string as above, retrieving additional 614 clinical trials, 203 of with results including 116 Phase 2 and 88 Phase 3 clinical trials. Among these, we then selected for the analysis studies including both ORR and PFS evaluated both locally and by BICR. A total of nine Phase 2 and eight Phase 3 clinical trials met the inclusion criteria. Among the newly found Phase 2 clinical trials, only four did not overlap with the search in the clinicaltrials.gov database, although only two could be included for the analysis (Table 1, trials number 16 and 17). In this regard, the EudraCT 2013-000311-25 (NCT01915589, with no results) and the EudraCT 2013-000241-39 evaluating the efficacy of refametinib in the hepatocellular carcinoma were excluded since it was not possible to calculate a discrepancy index for the ORR. In addition, the Phase 2 clinical trial number 16 (Table 1), EudraCT 2013-005486-39, was linked to two different trials in the US database, namely, the NCT02108652 and NCT02951767 clinical trials, including results from two different cohorts of patients. Both trials are reported in Table 1 as number 16. For the newly found Phase 3 clinical trials, only three did not overlap with those found in the clinicaltrials.gov database. The total number of Phase 3 trials considered for the analysis is therefore 21.

**TABLE 1:** Main characteristics of Phase 2 clinical trials selected for the analysis.

	Trial registration number (First posted date)	Intervention model/masking	Tumor type	Treatment arm(s)	Number of patients	Primary endpoint	Cycle length	Timing of scans	Time frame (primary endpoint)	DI ORR	DI PFS	Reference
1	NCT00875667	Parallel assignment/none (open label)	Relapsed or refractory mantle cell lymphoma	E: lenalidomide	E: 170	PFS per BICR and LI^a^	E: 28 days	Every 2 cycles for 6 months; every 90 days thereafter until disease progression	Median follow-up of 15.9 months (IQR 731.7) for all patientsa	E: 1.15	E: 0.99	Trněný et al. (2016)
	(3 Apr 2009)			AC: investigator’s choice single agent	AC: 84		AC: variable according to different regimens			AC: 2.09	AC: 1.04	
2	NCT01685060 (13 Sep 2012)	Single Group Assignment/None (Open Label)	Non-small-cell lung cancer	Ceritinib (LDK378)	140	ORR per LI	28 days	Every 8 weeks	Up to 24 weeks	1.14	0.78	Hida et al. (2017)
	EudraCT 2012-003432-24 (12 Apr 2017)											
3	NCT01685138 (14 Sep 2012)	Single group assignment/none (open label)	Non-small-cell lung cancer	Ceritinib (LDK378)	124	ORR per LI	28 days	Every 8 weeks	Up to 5 years	1.06	0.86	Nishio et al. (2020)
4	NCT01708174 (16 Oct 2012)	Single group assignment/none (open label)	Medulloblastoma	Sonidegib (LDE225) and TMZ	16	ORR per BICR	28 days	NA	>3 years	1.33	1	Clinicaltrials.gov identifier NCT01708174, 2012
5	NCT01984242 (14 Nov 2013)	Parallel assignment/none (open label)	Renal cell carcinoma	ITT	ITT	PFS per BICR	6 weeks	Every 12 weeks ± 5 days after cycle 1	Up to ∼2.75 years	ITT	ITT	McDermott et al. (2018)
				E1: atezolizumab + bevacizumab	E1: 101		E(1–2): one infusion every 3 weeks			E1: 1.09	E1: 0.95	
	EudraCT 2013-003167-58 (29 Oct 2017)			E2: atezolizumab	E2: 103		AC: Day 1–28 of each cycle			E2: 0.92	E2: 0.90	
				AC: sunitinib	AC: 101					AC: 1.14	AC: 0.93	
6	NCT02031458 (9 Jan 2014)	Single group assignment/none (open label)	Non-small-cell lung cancer	Atezolizumab	Cohort 1: 139	ORR per BICR	3 weeks	Every 6 weeks for 12 months; every 9 weeks thereafter	Up to 16 months	Cohort 1: 1.15	Cohort 1: 1.29	Peters et al. (2017)
				Cohort 1: first line	Cohort 2: 267					Cohort 2: 1.09	Cohort 2: 1.00	
	EudraCT 2013-003330-32 (1 July 2016)			Cohort 2: second line	Cohort 3: 253					Cohort 3: 1.05	Cohort 3: 1.07	
				Cohort 3: third line and beyond								
7	NCT02040870 (20 Jan 2014)	Single group assignment/none (open label)	Non-small-cell lung cancer	Ceritinib (LDK378)	103	Others	28 days	Every 8 weeks	40 months	1.30	1.89	Wu et al. (2020)
8	NCT02336451 (13 Jan 2015)	Parallel assignment/ none (open label)	ALK-positive Non-small-cell lung cancer	Ceritinib^c^ (LDK378)	Arm 1: 42	ORR per LI	28 days	At week 8; every 8 weeks thereafter	43 months	Arm 1: 1.50	Arm 1: 1.44	Chow et al. (2019)
					Arm 2: 40					Arm 2: 2.00	Arm 2: 1.02	
	EudraCT 2014-000578-20 (22 Feb 2020)				Arm 3: 12					Arm 3: 1.50^b^	Arm 3: NA^b^	
					Arm 4: 44					Arm 4: 0.96	Arm 4: 1.03	
					Arm 5: 18					Arm 5: 1.50	Arm 5: 1.44	
9	NCT00117637 (8 July 2005)	Parallel assignment/none (open label)	Renal cell carcinoma	E: first sorafenib (BAY43-9006) 400 mg then 600 mg	E: 97	PFS per BICR	NA	Every 8 weeks	∼15 months	E: 4.19	E: 0.98	Escudier et al. (2009)
				AC: first interferon then sorafenib 400 mg	AC: 92					AC: 1.75	AC: 1.25	
10	NCT01943864 (September 17, 2013)	Single group assignment/none (open label)	Advanced or metastatic biliary tract cancers in Japanese	Trametinib	20	Others	NA	Every 8 weeks	Up to week 37	NA^b^ (ORR per LI: 0)	1^b^	Ikeda et al. (2018)
11	NCT00679211 (16 May 2008)	Single group assignment/none (Open Label)	Metastatic breast cancer	Trastuzumab emtansine (Kadcyla)	110	ORR per BICR	3 weeks	Every other cycle by LI and retrospectively by double-reader BICR as needed	∼9 months	1	0.797	Krop et al. (2012)
12	NCT02674061 (4 Feb 2016)	Parallel assignment/none (open label)	Advanced recurrent ovarian cancer	Pembrolizumab (MK-3475)	Cohort A: 285	ORR per BICR (in all cohorts A and B and subgroup cohorts PDl-L1+)	3 weeks	Every 9 weeks for the first 54 weeks; every 12 weeks thereafter	Up to ∼43 months	Cohort A: 0.864	Cohort A: 1	Matulonis et al. (2019)
				Cohort A (0–2 prior lines of treatment)	Cohort B: 91					Cohort B: 0.889	Cohort B: 1	
				Cohort B (3–5 prior lines of treatment)								
13	NCT03525678 (16 May 2018)	Parallel assignment/none (open label)	Multiple myeloma	Belantamab mafodotin (GSK2857916)	Arm 1: 97	ORR per BICR	3 weeks	Every 3 weeks (radiography for skeletal lytic lesions, but it is not specified in the timing)	Up to 48 weeks	Arm 1: 0.968	Arm 1: 0.759	Lonial et al. (2020)
	EudraCT 2017-004810-25 (26 Apr 2020)			Arm 1 (2.5 mg/kg frozen liquid, FL)	Arm 2: 99					Arm 2: 0.912	Arm 2: 0.776	
				Arm 2 (3.4 mg/kg FL)	Arm 3: 25					Arm 3: 1.083^b^	Arm 3: NA^b^	
				Arm 3 (3.4 mg/kg lyophilized powder, LP)								
14	NCT02576990 (15 Oct 2015)	Single group assignment/none (open label)	Mediastinal large B-cell lymphoma (rrMLBCL)	Pembrolizumab (MK-3475)	Arm 1: 53	ORR per BICR	3 weeks	At week 12 and then every 12 weeks	Up to ∼27 months	Arm 1: 0.916	Arm 1: 0.782	Armand et al., 2019; Armand et al., 2020
			Richter syndrome (rrRS)	Arm 1 (rrMLBCL)	Arm 2: 23					Arm 2: 0.331	Arm 2: 1.125	
				Arm 2 (rrRS)								
15	NCT01660451 (8 Aug 2012)	Parallel assignment/none (open label)	Non-Hodgkin’s lymphoma (NHL)	Copanlisib (BAY80-6946)	Part A (indolent): 33	ORR per BICR	28 days	Every two cycles during year 1; every three cycles during year 2; every six cycles during year 3	Up to 16 weeks of treatment	Part A (indolent): 1.072	Part A (indolent): 0.952	Dreyling et al. (2017)
				Part A (indolent NHL/CLL)	Part A (aggressive): 51					Part A (aggressive): 1.154	Part A (aggressive): 1	Panayiotidis et al. (2021)
				Part A (aggressive NHL)	Part B: 142					Part B: 0.869	Part B: 0.971	
				Part B (indolent B-cell NHL)								
16	EudraCT 2013-005486-39 (28 July 2016) NCT02108652 (9 Apr 2014)	Single group assignment/none (open label)	Urothelial bladder cancer	Atezolizumab Cohort 2: second-line or beyond treatment	Cohort 2: 310	ORR per LI and BICR	21 days	Every 9 weeks for the first 12 months; every 12 weeks thereafter	Up to maximum length of follow-up of 24.5 months	1.04	1	Perez-Gracia et al. (2018)
	NCT02951767 (1 Nov 2016)			Cohort 1: drug naïve	Cohort 1: 119					1.11	1.55	Balar et al. (2017)
17	EudraCT 2013-002737-38 (28 Apr 2021) NCT02183870 (no results) (8 Jul 2014)	Single group assignment/	Lung cancer	Crizotinib	Per protocol: 30	ORR per LI	Continuous daily dosing	Every 6 weeks	NA	Per protocol: 0.959	Per protocol: 0.97	Michels et al. (2019)
		none (open label)	Adenocarcinoma NSCLC (ROS1 +)		ITT: 34					ITT: 0.959^b^	ITT: NA^b^	

Trials that were included in our initial analysis (Dello Russo et al., 2021) are highlighted in gray. IQR, interquartile range; ITT, intention to treat population; ORR, objective response rate; PFS, progression-free survival; BICR, blind independent central review; LI, local investigator; rrMLBCL, relapsed or refractory primary mediastinal large B-cell lymphoma; rrRS, relapsed or refractory richter syndrome; DI, discrepancy index; NHL, non-Hodgkin’s lymphoma; CLL, chronic lymphocytic leukemia; mRECIST, modified response evaluation criteria in solid tumors; PFI/TFI, platinum-free interval/treatment-free interval; PrALKi, previous ALK inhibitors’ treatment; PrBRad, previous brain radiations; RECIST, response evaluation criteria in solid tumors.

a The Primary outcome for this trial was PFS per BICR (up to data cutoff date, 7 March 2014) and LI (at final analysis, up to study discontinuation of 09 October 2018, (median follow up of 285 weeks). The data included in our analysis refer to the primary analysis cutoff date for both LI and BICR (**Trněný et al., 2016**). The same DIs are calculated by comparing the values obtained by LI at final analysis *versus* BICR primary analysis (Dello Russo et al., 2021).

b Not included in the present analysis since the specific arm/group was missing the DI for one of the outcomes (either ORR or PFS).

c Treatment arms were the followings: Arm 1 (PrALKi = Yes; PrBRad=Yes); Arm 2 (PrALKi=Yes; PrBRad=No); Arm 3 (PrALKi=No; PrBRad=Yes); Arm 4 (PrALKi=No; PrBRad=No); Arm 5 (leptomeningeal carcinomatosis). In Arm 1–4, no evidence of leptomeningeal carcinomatosis.

### Results From Phase 2 Clinical Studies

As summarized in Table 1, a total of 17 clinical Phase 2 clinical trials were selected for the current analysis, including nine studies, highlighted in gray, considered in our previous analysis (Dello Russo et al., 2021). All the trials reported in Table 1 had an open-label design and mostly (10/17, 58.8%) a single group assignment. Among the remaining studies with parallel group assignment, only two included an active comparator group, that is, the NCT00875667 (n. 1) clinical trial (Trněný et al., 2016), studying the efficacy of lenalidomide *versus* chemotherapy as per investigator’s choice in patients with mantle cell lymphoma after previous treatment failure, and the NCT01984242 (n. 5) clinical trial (McDermott et al., 2018), testing the efficacy of atezolizumab alone or in combination with bevacizumab *versus* sunitinib in patients affected by advanced renal carcinoma. All the other trials with parallel group assignment tested the same treatment in different subgroups or different schedule of administration of the same drug or drug combination. Therefore, all these studies substantially lacked a comparator arm. Interestingly, both the NCT00875667 and the NCT01984242 clinical trials, with the active comparator arm, included the PFS per BICR as primary endpoint. The latter was also found as the primary outcome in the NCT00117637 (n. 9) clinical trial (Escudier et al., 2009), testing the efficacy of sorafenib in combination with interferon-α (IFN-α) in two different schedules of administration, that is, Cohort 1 (Sorafenib First) and Cohort 2 (IFN-α First). With respect to the primary endpoint, the majority of the selected trials (7/17, 41%) included the evaluation of ORR per BICR, whereas only four (23.5%) trials reported the ORR per LI as the primary outcome. Interestingly, all these four trials were set to assess the efficacy of ALK1 inhibitors (three trials focused on ceritinib and one on crizotinib) in the context of non-small-cell lung cancer. In these trials, tumor assessment was performed every 8 weeks with a median time to first drug response of approximately of 2.0 months by BICR *versus* 2.3 months per LIs, which indicates a rapid pattern of tumor response to these inhibitors.

To evaluate the concordance between the two evaluations in this set of Phase 2 clinical trials, a discrepancy index (DI) was calculated for both the ORR (expressed as % of patients with CR and PR over the total number of the enrolled patients) and the PFS (considering the median PFS time for each treatment group) (Table 1). The DI was used in our previous analyses in order to assess differences between the two evaluations (Dello Russo et al., 2020; 2021). It is calculated as the ratio between the LI evaluation over the corresponding independently assessed endpoint, with a DI >1 indicating that the investigator was “more optimistic” and a DI <1 indicating the opposite, that the “blinded reviewer was more optimistic.” Among all these studies, the average DI for the ORR was 1.24 (95% CI, 1.005–1.478, *n* = 31) and the average DI for PFS was 1.05 (95% CI, 0.958–1.141). These data suggest that the LIs tend to overestimate the ORR, with a +24% of discrepancy which is little over what observed in our previous analysis (average + 17.5% of positive responses, Dello Russo et al., 2021). In line with previous data, we found a substantial agreement between the two evaluations for the PFS in these Phase 2 clinical trials. Interestingly, in the abovementioned four clinical trials on ALK inhibitors having the ORR per LI as the primary outcome, we found an average DI of 1.19 for both the ORR and the PFS which suggest a +19% overestimation of positive effects by LIs in comparison to BICR on both endpoints.

In Table 2, we summarized the results for eight different trials data on the time to first treatment response (TTFR), the time to progression (TTP) and the median PFS. From the comparison of these different endpoints, it emerges that the average time to observe a tumor response to treatment is relatively shorter that the time to reach progression. This is an expected finding, which implies that a reduced number of scans are indeed necessary to observe the response to treatment, therefore explaining in part the higher variability observed in the assessment of ORR locally and centrally. With time and the evaluation of multiple scans, the variability among the two evaluations tends to be reduced. Moreover, we can also hypothesize that it is more difficult to detect a response to treatment particularly in term of distinction between PR and stable disease in comparison to disease progression, which may further contribute to the higher discrepancy observed for the ORR among the two evaluations.

**TABLE 2:** Comparison between time to first response (TTFR), time to disease progression (TTP), and median PFS time in Phase 2 clinical trials.

	Trial registration number	Treatment arm(s)	Timing of scans	Number of patients (TTFR)	TTFR per LI (months, 95% CI)	TTFR per BICR (months, 95% CI)	Number of patients (PFS)	Time to progression (TTP) per LI (months, 95% CI)	Time to progression (TTP) per BICR (months, 95% CI)	Median PFS per LI (months, 95% CI)	Median PFS per BICR (months, 95% CI)
1	NCT00875667	E: lenalidomide	Every two cycles for 6 months; every 90 days thereafter until disease progression^a^	E: 170	E: 5.5 (3.9 – 5.9)	E: 4.3 (3.9 – 11.5)	E: 170	E: 9.1 (5.8 − 14.1)	E: 9.1 (5.6 – 12.2)	E: 8.6 (5.6 – 12.1)	E: 8.7 (5.5 – 12.1)
		AC: investigator’s choice single agent		AC: 84	AC: 9.2 (5.9 – NR)	AC: NR	AC: 84	AC: 5.7 (3.7 − 8.5)	AC: 5.7 (3.7 – 6.9)	AC: 5.4 (3.6 – 7.7)	AC: 5.2 (3.7 – 6.9)
2	NCT01685060 - EudraCT 2012-003432-24	Ceritinib (LDK378)	Every 8 weeks	LI: 57	3.0 (SD, 3.54)	2.2 (SD, 1.44)	140	NA	NA	5.8 (5.4–7.6)	7.4 (5.6–10.9)
				BICR: 50							
3	NCT01685138	Ceritinib (LDK378)	Every 8 weeks	LI: 84	2.5 (SD, 2.66)	2.2 (SD, 1.22)	124	NA	NA	16.6 (11.0–23.2)	19.4 (10.9–29.3)
				BICR: 79							
7	NCT02040870	Ceritinib (LDK378)	Every 8 weeks	LI: 43	1.90 (1.6–12.9)	1.80 (1.6–3.7)	103	NA	NA	7.2 (4.1–7.5)	3.8 (3.6–5.6)
				BICR: 33							
8	NCT02336451	Ceritinib (LDK378)	At week 8; every 8 weeks thereafter	**LI**	Arm 1: 1.87 (1.7–9.3)	Arm 1: 2.00 (1.7 – 12.9)	**LI**	NA	NA	Arm 1: 7.2 (3.3–10.9)	Arm 1: 5.0 (3.3–9.1)
				Arm 1: 15	Arm 2: 2.00 (1.7–9.3)	Arm 2: 1.76 (1.6–1.9)	Arm 1: 32			Arm 2: 5.6 (3.6–9.2)	Arm 2: 5.5 (3.6–7.3)
				Arm 2: 12	Arm 3: 1.82 (1.2–30.1)	Arm 3: 1.82 (1.7–26.5)	Arm 2: 35			Arm 3: NR (1.0 - NR)	Arm 3: 15.5 (1.0 - NR)
				Arm 3: 6	Arm 4: 1.81 (1.3–3.7)	Arm 4: 1.81 (1.3–22.0)	Arm 3: 6			Arm 4: 7.9 (5.5–9.4)	Arm 4: 7.7 (5.5–9.7)
				Arm 4: 26	Arm 5: 1.91 (1.8–3.6)	Arm 5: 1.86 (1.8–1.9)	Arm 4: 33			Arm 5: 5.2 (1.6–7.2)	Arm 5: 3.6 (1.6–5.4)
				Arm 5: 3			Arm 5: 14				
				**BICR**			**BICR**				
				Arm 1: 10			Arm 1: 34				
				Arm 2: 6			Arm 2: 36				
				Arm 3: 4			Arm 3: 8				
				Arm 4: 27			Arm 4: 33				
				Arm 5: 2			Arm 5:14				
9	NCT00117637	Sorafenib (BAY43-9006) + interferon	Every 8 weeks	**LI**	E: 3.5 (1.6–11.1)	E: 1.8 (1.7–3.7)	E: 97	NA	NA	E: 5.6 (5.4–7.5)	E: 5.7 (5.0–7.4)
				E: 21							
				AC: 14	AC: 5.4 (1.2–18.3)	AC: 5.4 (3.7–11)	AC: 92			AC: 7.0 (5.4–8.8)	AC: 5.6 (3.7–7.4)
				**BICR**							
				E: 5							
				AC: 8							
10	NCT01943864	Trametinib	Every 8 weeks	LI: 0	NA	20.1 weeks	20	NA	NA	10.6 weeks (4.6–12.1)	10.6 weeks (4.6–12.7)
				BICR: 1							
13	NCT03525678 (EudraCT 2017-004810-25)	Belantamab mafodotin (GSK2857916)	Every 3 weeks (radiography for skeletal lytic lesions, but it is not specified in the timing)	**LI**	Arm 1: 1.4 (0.8–2.1)	Arm 1: 1.4 (0.8–2.1)	Arm 1: 97	Arm 1: 2.3 (0.8 - NR)	Arm 1: 3.0 (0.9 - NR)	Arm 1: 2.2 (0.8 - NR)	Arm 1: 2.9 (0.9 - NR)
				Arm 1: 29	Arm 2: 1.5 (0.9–3.0)	Arm 2: 1.4 (0.8–2.8)	Arm 2: 99	Arm 2: 4.2 (1.3 - NR)	Arm 2: 5.8 (0.9 - NA)	Arm 2: 3.8 (1.1 - NR)	Arm 2: 4.9 (0.9 - NR)
				Arm 2: 31	Arm 3: 0.9 (0.8–1.0)	Arm 3: 0.9 (0.8–1.6)	Arm 3: 25	Arm 3: 4.3 (2.1 - NR)	Arm 3: NA (2.2 - NA)	Arm 3: 4.3 (2.1 - NR)	Arm 3: NA (2.2 - NR)
				Arm 3: 13							
				**BICR**							
				Arm 1: 30							
				Arm 2: 34							
				Arm 3: 12							

Trials that were included in our initial analysis (Dello Russo et al., 2021) are highlighted in gray. ITT, intention to treat population; NA, not available; NR, not reached; ORR, objective response rate; PFS, progression-free survival; BICR, blind independent central review.

a Data included in the table refer to the analysis at data cutoff date, 7 March 2014 (Trněný et al., 2016).

### Results From Phase 3 Clinical Studies

A total of 21 Phase 3 clinical trials, including both ORR and PFS evaluated locally and centrally were found (Table 3). These included one trial, the NCT00075270 (n. 3) (Di Leo et al., 2008), in which the time to disease progression (TTP) per LI and BRIC was included as primary endpoint and considered in place of the PFS for the present evaluation. The trial NCT01287741 (n. 15) only included the HR for the evaluation of PFS by LI and BICR (Vitolo et al., 2017). Both trials were also included in our previous analysis (Dello Russo et al., 2020), therefore kept in the present analysis. The majority of these studies (17/21, 81%) had an open-label design and (10/21, 47.6%) included the evaluation of PFS per BICR as primary endpoint. In this regard, only six studies out of 21 (28.6%) included the evaluation of PFS per LI as the primary outcome, whereas three studies (14.2%) had both evaluations as the primary endpoint.

**TABLE 3:** Main characteristics of Phase 3 clinical trials selected for the analysis.

	Trial registration number (first posted date)	Intervention model/masking	Tumor type	Treatment arm(s)	Number of patients	Primary endpoint	Cycle length	Timing of scans	Time frame (primary endpoint)	DI ORR	DI PFS	Reference
1	NCT00069108 (September 18, 2003)	Parallel assignment/none (open label)	Colorectal cancer	E: XELOX	E (ITT): 313	PFS per LI	E: 3 weeks up to eight cycles	Every 6 weeks (+ within 2 weeks of study completion, withdrawal or treatment discontinuation)	Up to 3 years	E (ITT): 1.50	E (PP): 0.92	Rothenberg et al. (2008)
				AC: FOLFOX-4	AC (ITT): 314							
					E (PP): 251		AC: 2 weeks up to 12 cycles			AC (ITT): 1.33	AC (PP): 1.04	
					AC (PP): 314							
2	NCT02370498 (25 Feb 2015)	Parallel assignment/none (open label)	Gastric adenocarcinoma	E: pembrolizumab	E (all): 296AC (all: 296	PFS (and OS) per BICR in PD-L1+ patients	E: 21 days	Every 6 weeks	Up to 30 months	E (all): 1.10	E (all): 1.07	Shitara et al. (2018)
			Gastroesophageal junction adenocarcinoma	AC: paclitaxel	E (PD-L1+):					AC (all): 1.22	AC (all): 0.78	
					196		AC: 28 days			E (PD-L1+): 1.09^a^	E (PD-L1+): 1.07^a^	
					AC (PD-L1+): 199					AC (PD-L1+: 1.15^a^	AC (PD-L1+: 0.76^a^	
3	NCT00075270 (9 Jan 2004)	Parallel assignment/	Metastatic breast cancer	E: lapatinib (+ paclitaxel)	E: 291	TTP per LI and BICR	E: 3 weeks	For efficacy 9 weeks after study entry, at 12-week intervals, and at treatment end. For survival at 12-week intervals	Average 26 weeks	E: 1.31	E: 0.86^b^	Di Leo et al. (2008)
		double (participant, investigator)		AC: placebo (+ paclitaxel)	AC: 288		AC: 3 weeks			AC: 1.35	AC: 0.88^b^	
4	NCT01120184 (10 May 2010)	Parallel assignment/	Breast cancer	AC: trastuzumab + taxane	PFS	PFS per BICR	3 weeks (except paclitaxel every 1 week)	Every 9 weeks for 81 weeks, then every 12 weeks thereafter, and/or up to 42 days after last dose	Up to 48 months	AC: 1.02	AC: 0.91	Perez et al. (2017)
		triple (participant, investigator, and outcomes assessor)		E1: trastuzumab emtansine + placebo	AC: 365					E1: 1.08	E1: 1	
				E2: trastuzumab emtansine + pertuzumab	E1: 367					E2: 1.05	E2:0.97	
					E2: 363							
					ORR							
					AC: 287							
					E1: 303							
					E2: 299							
5	NCT00689936 (4 Jun 2008)	Parallel assignment/	Multiple myeloma (previously untreated; stem cell transplant ineligible)	Arm 1: lenalidomide + low-dose DEX (until disease progression)	Arm 1: 535	PFS per BICR and LI	4 weeks	After each treatment cycle and every	PFS by BICR: median follow-up time of 17.1 months	Arm 1: 1.07	Arm 1: 1.02	Benboubker et al. (2014)
		none (open label)		Arm 2: lenalidomide + low-dose DEX (18 cycles)	Arm 2: 541			28 days during the follow-up phase	PFS by BICR: median follow-up time of 17.7 months	Arm 2: 1.07	Arm 2: 1.01	
				Arm 3/AC: melphalan + prednisone + thalidomide	Arm 3/AC: 547					Arm 3/AC: 1.08	Arm 3/AC: 1.03	
6	NCT01360554 (25 May 2011)	Parallel assignment/	Non-small-cell lung cancer	E: dacomitinib (PF-00299804) + placebo (erlotinib)	All population	PFS per BICR and	28 days (continuous oral daily dosing)	At the end of cycles 2, 3, and 4, then every other cycle	Median follow-up of 7.1 months, until disease progression	E: 1.13	E: 0.73	Ramalingam et al. (2014)
		quadruple (participant, care provider, investigator, and outcomes assessor)		AC: erlotinib + placebo (PF-00299804)	E: 439	PFS in KRAS wild-type patients				AC: 1.29	AC: 0.76	
					AC: 439							
7	NCT01774721 (24 Jan 2013)/EudraCT	Parallel assignment/	Non-small-cell lung cancer with EGFR-activating mutations	E: dacomitinib (PF-00299804)	E: 227	PFS per BICR	28 days (continuous oral daily dosing)	At the end of cycles 1–2, then at	Up to 48 months	E: 1.01	E: 1.13	Wu et al. (2017)
	2012-004977-23 (25 Oct 2018)	none (open label)		AC: gefitinib	AC: 225			every other cycle		AC: 0.98	AC: 1.20	
8	NCT02604342 (13 Nov 2015)/EudraCT	Parallel assignment/	Non-small-cell lung cancer	E: alectinib	E: 79	PFS per LI	3 weeks (alectinib: continuous oral twice daily dosing)	Every 6 weeks	Up to 33 months	E: 1.40	E: 1.35	Novello et al. (2018)
	2015-000634-29	none (open label)		AC: premetrexed/	AC: 40					AC: 0.22	AC: 0.875	
				docetaxel								
9	NCT01245062 (22 Nov 2010)	Crossover assignment/	Melanoma	E: trametinib (GSK1120212)	BRAF V600E + w/o brain metastasis	PFS in BRAF V600E+ w/o brain metastasis per BICR and LI	3 weeks (trametinib: continuous dosing)	At weeks 6, 12	Average of 20.3 months	BRAF V600E+ w/o brain metastasis	BRAF V600E+ w/o brain metastasis	Flaherty et al. (2012)
		none (open label)		AC: dacarbazine or paclitaxel	E: 178			21, and 30; then, every 12 weeks		E: 1.30	E: 0.92	
					AC: 75					AC: 2.33	AC: 0.88	
10	NCT02718417 (24 Mar 2016) EudraCT	Parallel assignment/	Ovarian cancer	AC: chemotherapy then observation	AC: 335	PFS per BICR	Chemotherapy: 3 weeks	After three cycles and at completion of chemotherapy; then, every 12 weeks during maintenance	Maximum duration of 27 months	AC: 0.914^a^	AC: NA^a^	Monk et al. (2021)
	2015-003239-36	none (open label)		E1: chemotherapy then avelumab in maintenance	E1: 332		Avelumab: 2 weeks			E1: 0.852	E1: 0.821	
				E2: chemotherapy in combination with avelumab then avelumab in maintenance	E2: 331					E2: 0.864	E2: 0.890	
11	NCT00083889 (4 Jun 2004)	Parallel assignment/	Renal cell carcinoma	AC: IFNα	AC: 375	PFS per BICR and LI	AC: 3 weeks	At day 28 of cycles 1 through 4, and every two cycles thereafter until the end of treatment	Duration of treatment phase	AC: 1.50	AC: 1.00	Motzer et al. (2007)
		none (open label)		E: sunitinib (SU011248)	E: 375		E: 6 weeks			E: 1.19	E: 0.99	
12	NCT02421588 (April 20, 2015) EudraCT	Parallel assignment/	Ovarian cancer (platinum resistant)	E (Arm A): lurbinectedin (PM01183)	E: 221	PFS per BICR	E: 3 weeks	Every 8 weeks	Up to 3 years	E: 1.09	E: 1.10	Gaillard et al. (2021)
	2014–005251-39 (17 Oct 2019)	none (open label)		AC (Arm B): pegylated liposomal doxorubicin or topotecan	AC: 221		AC: pegylated liposomal doxorubicin (4 weeks)			AC: 1.31	AC: 1.00	
							topotecan (3 weeks)					
13	NCT03052608 (14 Feb 2017)	Parallel assignment/	Non-small-cell lung cancer	E: lorlatinib	E: 149	PFS per BICR	28 days	Every 8 weeks (±1 week)	Up to 33 months	E: 1.06^a^	E: NA^a^	Shaw et al. (2020)
		none (open label)		AC: crizotinib	AC: 147					AC: 1.07	AC: 0.98	
14	NCT01102426 (13 Apr 2010)	Parallel assignment/	Relapsed/refractory multiple myeloma	E: plitidepsin + dexamethasone	E: 171	PFS per BICR	4 weeks	NA	Up to 5 years	E: 1.31	E: 1.12	Spicka et al. (2019)
		none (open label)		AC: dexamethasone	AC: 84					AC: 0.33	AC: 0.65	
15	NCT01287741^c^	Parallel assignment/	Diffuse large B-cell lymphoma	E: obinutuzumab + chemotherapy	E: 712	PFS per LI	21 days	4–8 weeks (CT) or 6–8 weeks (FDG-PET) after the last treatment or sooner in the case of early discontinuation	LE up to approximately 6.5 years	E: 0.999^a^	NA^a^	Vitolo et al. (2017)
	(1 Feb 2011) EudraCT 2010-024194-39	none (open label)		AC: rituximab + chemotherapy	AC: 706				BICR: up to approximately 4 years and 9 months	AC: 0.992^a^		
	(23 Apr 2017)											
16	NCT02580058^d^	Parallel assignment/	Ovarian cancer	E1: avelumab	E1: 188	PFS per	Avelumab: 2 weeks; doxorubicin: 4 weeks	MRI or CT scans every 8 weeks	Up to 30 months	E1: 1.43	E1: 1.00	Pujade-Lauraine et al. (2021)
	(20 Oct 2015)	none (open label)		E2: avelumab plus pegylated liposomal doxorubicin (PLD)	E2: 188	BICR and OS				E2: 1.40	E2: 1.27	
				AC: PLD alone	AC: 190					AC: 2.26	AC: 1.06	
17	NCT02603432^d^	parallel assignment/	Urothelial cancer	E (Arm A): avelumab plus best supportive care (BSC)	E (Arm A): 350	OS	4 weeks	Every 8 weeks for 12 months and then every 12 weeks	Up to 41 months at the time of final analysis	E (Arm A): 1.27	E (Arm A): 1.5	Powles et al. (2020)
	(11 Nov 2015)	none (open label)		Arm B: best supportive care (BSC) alone	Arm B: 350					Arm B: 2.43	Arm B: 1.05	
18	EudraCT	Parallel assignment/	Untreated advanced renal cell carcinoma	AC: sunitinib	ITT:	PFS per LI in PD-L1 selected population	AC: 4 weeks on, 2 weeks off	At week 12, then every 6 weeks up to week 78, and then every 12 weeks	Up to approximately 24 months	ITT	ITT	Rini et al. (2019)
	2014-004684-20 NCT02420821 (20 Apr 2015)	none (open label)		E: atezolizumab + bevacizumab	AC: 461		E: 3 weeks			AC: 1.064	AC: 1.012	
					E: 454					E: 1.099	E: 1.167	
19	EudraCT 2010-024132-41	Parallel assignment/	Non-Hodgkin’s lymphoma	E: obinutuzumab + chemotherapy	Follicular lymphoma population	PFS per LI in the follicular lymphoma population	21 or 28 days	After three cycles (bendamustine treated) or four cycles (CHOP or CVP) and on the completion of induction therapy; every 2 months for 2 years; then, every 3–6 months, with CT performed every 6–12 months, until progression or withdrawal from the trial	Up to ∼4 years and 7 months	E: 0.957	E: 1.15	Hiddemann et al. (2018); Marcus et al. (2017)
	(16 Mar 2017)	none (open label)		AC: rituximab + chemotherapy	E: 601					AC: 0.970	AC: 1.08	
	NCT01332968 (11 Apr 2011)				AC: 601							
20	EudraCT	Parallel assignment/	Advanced BRAFV600 wild-type melanoma	E: cobimetinib + atezolizumab	E: 222	PFS per BICR	Every 8 weeks through 80 weeks; then, every 12 weeks until progression	3 weeks	For approximately 16 months	E: 1.07	E: 1.02	Gogas et al. (2021)
	2016-004387-18^d^ (01 May 2020) NCT03273153 (6 Sep 2017)	none (open label)		AC: pembrolizumab	AC: 224					AC: 1.16	AC: 1.26	
21	NCT00789373 (First posted: 11 Nov 2008)	Parallel assignment/	Non-small-cell lung cancer	E: pemetrexed (maintenance)	E: 316/359	PFS per LI	Every other cycle (6 weeks [±1])	21 days	(Up to 19.3 months)	E: 1	E: 1.043	Paz-Ares et al. (2012)
		quadruple (participant, care provider, investigator, and outcomes assessor)		AC: placebo	AC: 156/180					AC: 1	AC: 1.088	

Trials that were included in our initial analysis (Dello Russo et al., 2021) are highlighted in gray. ITT, intention to treat population; ORR, objective response rate; PFS, progression-free survival; PD-L1, programmed cell death ligand 1; PP, per protocol; TTP, time to progression.

a Not included in the analysis because the subgroup of the whole population or the specific arm/group was missing the DI for one of the outcomes (either ORR or PFS).

b Time to progression was used for comparative analysis.

c Trial NCT01287741 is included in the table because HR values for PFS per LI and BICR were available for comparison. DI was calculated and included in a pooled analysis (*see* text).

d For these trials, DI based of the HR values for PFS was not available.

To assess the concordance between the two evaluations in this set of Phase 3 clinical trials, a discrepancy index (DI) was calculated for both the ORR (expressed as % of patients with CR and PR over the total of the enrolled patients) and the PFS (considering the median PFS time for each treatment group). Among all these studies, the average DI for the ORR was 1.20 (95% CI, 1.075–1.328, *n* = 42) and for PFS was 1.014 (95% CI, 0.963–1.065, *n* = 42). These data suggest that the LIs tend to overestimate the ORR, by a factor of +20% in the selected Phase 3 clinical trials, whereas there is a substantial agreement between the two evaluations for the PFS when considering the PFS median time.

In addition, for the ORR, we calculated the DI for the odd ratios (ORs), when reported (four trials), and for the PFS, the DI based on the ratio of HRs was calculated per type of evaluation for most of the trials. The DI calculated on the ORs for the ORR was 1.04, 95% CI 0.912–1.168, indicating a potential agreement when it comes to the evaluation of treatment effect among the two evaluations. With respect of the DI calculated on the HRs for PFS, out of 21 trials included in the present analysis, 13 were already considered in our previous study (Dello Russo et al., 2020), whereas six trials brought additional new data. On average, the DI calculated on the HRs from these six trials was lower than that from the previous observations (0.84, 95% CI 0.756–0.923, *n* = 6). However, by pooling these data with those of previous analysis, the average DI value was only slightly reduced, and 95% CI included the value 1 (0.958, 95% CI 0.91–1.01, *n* = 38).

As shown in Table 4, for three Phase 3 trials, it was possible to evaluate the TTFR together with the PFS. In line with data from Phase 2 clinical trials, the TTFR was shorter than the median PFS, with minimal differences observed in NCT02603432 (n. 17) trial evaluating the efficacy of avelumab in combination with best supportive care as maintenance therapy in patients with locally advanced or metastatic urothelial cancer with stable disease after completion of first-line platinum-containing chemotherapy (Powles et al., 2020). However, in this study only 34 responses out of 350 treated patients were observed in the experimental group in comparison to five responses out of 350 patients in the comparator arm by BICR. The LI reported a higher number of tumor responses in comparison to BICR. The fact that the ORR is often calculated based on a reduced number of responses may also contribute to the higher variability observed for the assessment of this endpoint by LIs and BICR.

**TABLE 4:** Comparison between time to first response (TTFR), time to disease progression (TTP), and median PFS in Phase 3 clinical trials.

	Trial registration number	Treatment arm(s)	Timing of scans	Number of patients (TTFR)	TTFR per LI (months, 95% CI)	TTFR per BICR (months, 95% CI)	Number of patients	Time to progression (TTP) per LI (months, 95% CI)	Time to progression (TTP) per BICR (months, 95% CI)	Median PFS per LI (months, 95% CI)	Median PFS per BICR (months, 95% CI)
5	NCT00689936	Arm 1: lenalidomide + low-dose DEX (until disease progression)	After each treatment cycle and every 28 days during the follow-up phase	Arm 1: 402	Arm 1: 1.8 (0.50–22.2)	Arm 1: 1.8 (0.7–22.2)	Arm 1: 535	NA	NA	Arm 1: 26.0 (20.7–29.7)	Arm 1: 25.5 (20.7–29.4)
		Arm 2: lenalidomide + low-dose DEX (18 cycles)		Arm 2: 397	Arm 2: 1.8 (0.8–34.8)	Arm 2: 1.8 (0.8–17.1)	Arm 2: 541			Arm 2: 21.0 (19.7–22.4)	Arm 2: 20.7 (19.4–22.0)
		Arm 3/AC: melphalan + prednisone + thalidomide		Arm 3/AC: 341	Arm 3/AC: 2.8 (1.2–56.3)	Arm 3/AC: 2.8 (1.3–49.7)	Arm 3/AC: 547			Arm 3/AC: 21.9 (19.8–23.9)	Arm 3/AC: 21.2 (19.3–23.2)
13	NCT03052608	E: lorlatinib	Every 8 weeks (±1 week)	E: 113	NA	E: 1.8 (1.7–1.9)	E: 149	NA	NA	E: NR (NR to NR)	E: NR (NR to NR)
		AC: crizotinib		AC: 85		AC: 1.8 (1.7–1.9)	AC: 147			AC: 9.1 (7.4–10.9)	AC: 9.3 (7.6–11.1)
17	NCT02603432	E (Arm A): avelumab plus best supportive care (BSC)	Every 8 weeks for 12 months and then every 12 weeks	LI	E (Arm A): 2.0 (1.8–22.2)	E (Arm A): 2.0 (1.7–16.4)	E (Arm A): 350	NA	NA	E (Arm A): 5.5 (4.2–7.2)	E (Arm A): 3.7 (3.5–5.5)
		Arm B: best supportive care (BSC) alone		E (Arm A): 43	Arm B: 1.9 (1.1–10.9)	Arm B: 2.0 (1.8–7.0)	Arm B: 350			Arm B: 2.1 (1.9–3.0)	Arm B: 2.0 (1.9–2.7)
				Arm B: 12							
				BICR							
				E (Arm A): 34							
				Arm B: 5							

Trials that were included in our initial analysis (Dello Russo et al., 2021) are highlighted in gray. ITT, intention to treat population; ORR, objective response rate; PFS, progression-free survival; BICR, blind independent central review; NA, not available; NR, not reached.

## Conclusion

Considering the review of available evidence carried out in this study, along with additional new evidence shown here for the first time, we can attempt to draw some overall conclusions:

✓ The additional analysis presented in this work confirmed that *1*) LIs tend to overestimate the ORR in comparison to BICR, whereas *2*) no significant differences are observed between LI and BICR concerning the assessment of PFS.
✓ The assessment of ORR is endowed with higher variability than that of PFS. Such larger variability is associated with various factors, including *1*) a limited number of measurements, compared to repeated measures with PFS; *2*) the time-to-response, which is a variable in ORR assessment, whereas PFS is always measured once a response is established; *3*) the time-to-response is in turn influenced by the type of treatment, with small molecules in general inducing faster responses than immunotherapies; *4*) some protocols may assess ORR at fixed times, while other may consider the *best response* to calculate ORR. Thus, a higher variability seems to be associated to a significant expectation bias.
✓ The analysis of ORR assessment in Phase 3 trials showed that, luckily, LIs tend to overestimate ORR compared to BICR both in experimental and control groups. Thus, by analyzing the data as ORs, the overestimations of control ORRs tend to counterbalance those of experimental ORRs, thereby reducing the gap between LI and BICR.
